# Physicochemical Aspects of Reaction of Ozone with Galactolipid and Galactolipid–Tocopherol Layers

**DOI:** 10.1007/s00232-014-9681-9

**Published:** 2014-05-27

**Authors:** Elżbieta Rudolphi-Skórska, Maria Filek, Maria Zembala

**Affiliations:** Department of Biochemistry, Biophysics and Biotechnology, Institute of Biology, Pedagogical University of Cracow, Podchorążych 2, Cracow, 30-084 Poland

**Keywords:** Galactolipids, Monolayers, Zeta potential, Oxidation, Protective action of α-tocopherol

## Abstract

The impact of reaction of galactolipids with ozone on the physicochemical properties of their monolayers was examined. In Megli and Russo (Biochim Biophys Acta, 1778:143–152, 2008), Cwiklik and Jungwirth (Chem Phys Lett, 486:99–103, 2010), Jurkiewicz et al. (Biochim Biophys Acta, 1818:2388–2402, 2012), Khabiri et al. (Chem Phys Lett, 519:93–99, 2012), and Conte et al. (Biochim Biophys Acta, 1828:510–517, 2013), the properties of layers formed from model mixtures composed of chosen lipids and selected oxidation products were studied, whereas in this work, question was raised as to how the oxidation reactions taking place *in situ* affect the physical properties of the galactolipid layers. So, set experiment should take into account the effect of all reaction products. The mechanical characteristics of monolayers of monogalactosyldiacyl-glycerol (MGDG) and digalactosyldiacylglycerol (DGDG) were determined by Langmuir trough technique, and the electrical properties of liposomes formed from these lipids by measuring their electrophoretic mobility. Considerable loss of galactolipid molecules forming monolayers was found at ozone concentrations (in aqueous medium) higher than 0.1 ppm with a stronger effect measured for MGDG. That goes along with the greater amounts of MDA found in the extracts of oxidized MGDG films compared with DGDG. Based on this, it was concluded that an additional galactose group present in DGDG molecules acts protectively under oxidative conditions. The surface tension of the solutions (of small volume) contacting the oxidized galactolipids films was significantly reduced, indicating the presence of soluble in polar media, surface active reaction products. The presence of α-tocopherol in mixtures with tested galactolipids at a molar ratio of lipid to tocopherol equal to 1.7:1 caused some inhibition of lipid oxidation, reducing the decrease of amount of lipid particles forming the monolayer. Here, also protective effect of α-tocopherol was greater for the MGDG compared to DGDG.

## Introduction

Biotic and abiotic factors including ozone action result in oxidative stresses in plant cells. High concentrations of ozone found mainly in urban areas represent a problem of developed and developing countries. Ozone concentration increases due to the photochemical oxidation of volatile organic compounds (VOCs) catalyzed by NO_*x*_ in the presence of sunlight (Sillman and He 2002; Thompson et al. 2003; Zhang et al. 2009). The local emission-related increase in ozone content in the atmosphere is subjected to cyclical changes throughout the day (Zhang et al. 2004). Ozone at high concentrations has a significant impact on health of organisms as well as on the functioning of entire ecosystems (McMichael et al. 2003). Chronic exposure of plants to ozone causes a considerable decrease in the efficiency of photosynthesis, reducing the production of biomass, and seeds.

Ozone has very high oxidizing ability which has placed this agent on the top of the list of oxidants when characterized by its redox potential. Being very reactive is unstable in various environments undergoing numerous, usually very fast reactions depending on media composition (Lovato et al. 2009). In bio-relevant systems, it reacts with unsaturated organic chemicals attacking multiple bonds. Mechanisms of oxidation include usually multiple reaction paths resulting in formation of various products (Frankel 1980; Porter et al. 1995; Fruhwirth et al. 2007; Megli and Russo 2008; Onyango 2012; Reis and Spickett 2012).

Breeding of plants resistant to stressors requires knowledge of mechanism of oxidative stress, which so far has not been fully recognized. Oxidative stress is associated with the overproduction of reactive oxygen species (ROS). ROS include: superoxide anion O_2_^•–^, hydrogen peroxide, H_2_O_2_, hydroxyl radical ^•^OH, and singlet oxygen. (Miller et al. 2010). In plant cells, the chloroplasts are the largest generator of intracellular ROS produced during photosynthesis (Asada 2006).

The membranes of chloroplasts are composed of about 50 % monogalactosyldiacyl-glycerol (MGDG), 30 % digalactosyldiacylglycerol (DGDG), 5–10 % sulfoquinovosyl diacylglycerol (SQDG), and 10 % phosphatidylglycerols (PG) (Dörmann and Hölzl 2009). Lipids of chloroplast membranes provide an environment for complex membrane proteins involved in photosynthesis. The presence of these lipids is essential for the fulfillment of specific biochemical functions by membranes, e.g., the significant loss of MGDG and DGDG found in mutants of *Arabidopsis* with a defective DGD and MGD synthase genes leads to striking defects in chloroplast ultrastructure which in consequence causes their abnormal operation (Jarvis et al. 2000; Kelly et al. 2003; Aronsson et al. 2008). At the molecular level, it was proved that the short-term exposure of plants to relatively large ozone concentrations (0.15–0.6 ppm) results in a significant reduction in the amount of galactolipids in the chloroplasts membranes and in a decrease of the fatty acid chains unsaturation (Harwood 1998). It was found (Filek et al. 2010a) that the aspect ratio of MGDG to DGDG in chloroplast envelopes of rape exposed to oxidative stress was changed. ROS excess generated under oxidative stress may cause lipid peroxidation, the measure of which may be an increase in the concentration of malondialdehyde (MDA) (Moore and Roberts 1998).

A balance between the production and destruction of ROS is maintained by protective systems which include enzymatic (catalase, peroxidase, superoxide dismutase) and non-enzymatic (hydrophilic such as ascorbic acid and glutathione and hydrophobic, e.g., tocopherols) antioxidants (Apel and Hirt 2004). Tocopherols—antioxidants synthesized and stored in the plastids—are involved in protection of the photosynthetic apparatus against the toxic action of ROS (Lushchak and Semchuk 2012). Tocopherol molecules can physically quench singlet oxygen but also chemically scavenge ^1^O_2_ and superoxide lipid radicals, protecting photosynthetic apparatus from peroxidation (Munné-Bosch and Alegre 2002; Munné-Bosch 2005). Tocopherol level depends on the stress intensity, physiological state of the plant, and the species sensitivity to stress increasing significantly under oxidizing conditions (Munné-Bosch 2005).

There are many studies focused on the direct action of ozone on lipids of animal cells—red blood cell membrane (Uppu et al. 1995), pulmonary surfactant (Kim et al. 2010; Ko et al. 2012). However, little research is done on lipids typical for plant cells. Therefore, the presented results concern the direct action of ozone on layers formed from MGDG and DGDG lipids typical for plant cells and the possible protective effect of α-tocopherol.

The aim of the paper was to determine how products of reactions of ozone with DGDG and MGDG modify physical properties of their monolayers. In papers: Megli and Russo (2008), Cwiklik and Jungwirth (2010), Jurkiewicz et al. (2012), Khabiri et al. (2012), Conte et al. (2013) the model mixtures composed of defined lipids and some chosen products of their oxidation were studied either experimentally or described by MD simulations. The effect of oxidized species presence on various properties of mixed model layers was determined. Unlike in these works, we addressed the question how the oxidation process taking place in situ influences the physical properties of the ozone-treated lipid layers.

The impact of ozone onto layers of α-tocopherol—effective antioxidant—present individually and in mixtures with studied lipids was also studied. This work is a continuation of the earlier research conducted for galactolipids extracted from plants grown under stress conditions (Gzyl-Malcher et al. 2010). Relatively high concentrations of tocopherol were used in presented studies to obtain measurable effects. However, some justification for chosen composition of lipid/tocopherol mixtures can be found in the literature (Atkinson et al. 2010), where protective ability of tocopherol, present in natural membranes at low global concentrations, was explained by hypothesis of its partition into membrane domains enriched in polyunsaturated phospholipids, increasing many-fold its concentration at such locations.

## Materials and Methods

### Materials

*Galactolipids* of known composition of fatty acid residuals (given by the supplier—Table 1): mono- (MGDG) and di-galactosyldiacylglycerol (DGDG) were from Avanti Polar Lipids Inc. (USA/Canada).

**Table 1:** Fatty acids residue distribution in studied samples of galactolipids (according to Avanti Polar Lipids Inc.)

Fatty acids residues	MGDG	DGDG
16:0	0	4.85
16:1	1.5	3.45
16:3	39.85	14.15
18:2	8.05	8.85
18:3	50.6	68.8

*Phosphatidic acids*-(1,2-dipalmitoyl-sn-glycero-3-phosphate 16:0) (DPPA) and (1,2-dioleoyl-sn-glycero-3-phosphate 18:1) (DOPA) were obtained from Sigma.

α-*Tocopherol* of ≥96 % purity—from Sigma.

KCl used in experiments was of chemical purity.

*Solvents* (chloroform, methanol) of chemical purity were from POCh (Poland).

Freshly deionized water was produced by HLP 5 apparatus Hydrolab (Poland).

1 mM KCl was used as a supporting electrolyte in all experiments.

### Methods

#### Methods of Ozonation and Determination of Ozone Concentration

Ozone generator FM 500 (prod. F-ma Grekos, Poland) supplied by air, working on the principle of corona discharge was used as a source of ozone. The yield of ozone production was in the range 200–500 mg/h. Gas mixture produced by ozone generator was bubbled through a scrubber containing sodium carbonate solution of high pH to remove nitrogen oxides formed simultaneously during the electric discharges. Solution of supporting electrolyte was saturated with ozone containing gas. Defined ozone concentrations were obtained by the appropriate dilution of the electrolyte saturated with ozone. In some preliminary experiments gas containing ozone was administrated (usually for 6 or 10 min) directly to the housing of Langmuir trough. Ozone concentration in solutions was determined by classical indygo method based on the decrease in absorbance of the indigo carmine (sodium indigodisulfonate) at *λ* = 287 nm (Bader and Hoigne 1981, 1982; Majewski 2012). Discoloration of the dye was calibrated versus direct ozone UV peak at 250 nm used as a primary standard with molar extinction coefficient equal to 3,200 M^−1 ^cm^−1^ (according to Sonntag and Gunten (2012)). As ozone content in aqueous solutions decreases very fast due to multiple reactions, samples of solutions for analysis were taken directly from Langmuir trough at the moment of lipid deposition.

#### Surface Pressure Isotherms

Surface pressure isotherms were obtained using Langmuir trough (Minitrough, KSV, Finland) of total surface area 243 cm^2^ with Pt-Wilhelmy plate used for surface tension detection. Measurements were taken at temperature of 25 °C at a constant rate of barrier movement equal to 5 mm/min which for most of experiments corresponded to the rate of area per molecule decrease being in the range 2–4 Å^2^ molecule^−1^ min^−1^.

#### Electrokinetic Measurements

For some systems, particle electrophoretic mobility was determined by dynamic light scattering technique using Malvern Zetasizer ZS apparatus. The values of mobility were converted to zeta potentials using Smoluchowski equation.

Thin layers of studied compounds were formed by evaporating (under argon) of their solution in chloroform wetting the walls of the round-bottomed glass cell. Dried film was next dispersed in a defined amount of 1 mM KCl solution by vortexing and ultrasonification. Under such conditions, DGDG formed liposomes, whereas α-tocopherol film was comminuted to form irregular particles.

Ozonized samples were obtained by 10 min exposition of dried lipid films to gas produced by ozone generator.

#### Surface Tension Measurements

Surface tensions of solutions contacting oxidized lipid and α-tocopherol films were determined by stalagmometric method from the mass of drop.

#### MDA Determination

The level of lipid peroxidation was evaluated by an amount of MDA formed in the oxidation reaction. The quantity of MDA was determined by measuring absorbance of MDA/TBA adduct (Sochor et al. 2012). Samples were added to 0.25 % TBA in 10 % trichloroacetic acid. The mixture was heated at 95 °C for 30 min and, after cooling, centrifuged at 10,000 rpm. The absorbance of supernatant was measured at 532 nm. The MDA content was calculated using the extinction coefficient equal to 155 mM^−1 ^cm^−1^.

## Results and Discussion

**Fig. 1:**
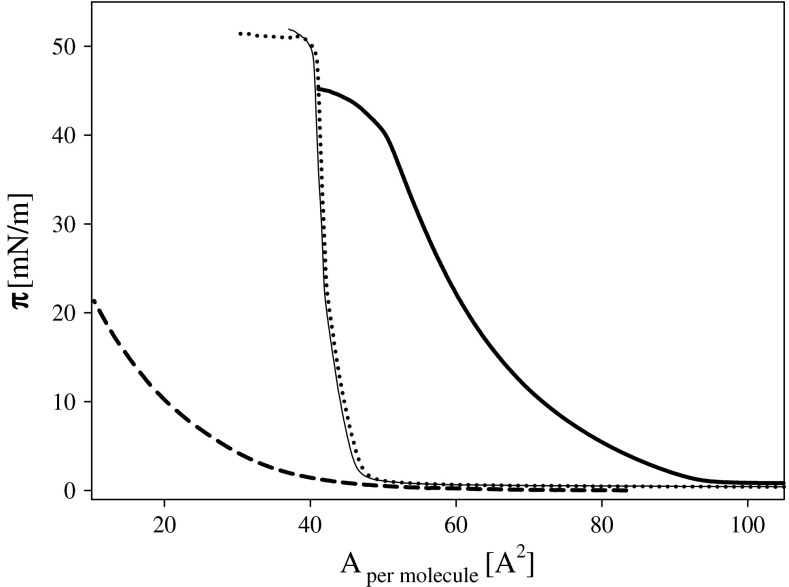
Surface pressure isotherms of PA layers deposited on 1 mM KCl solution when Langmuir trough chamber was filled with air: DPPA *thin solid line*, DOPA 18:1 *thick solid line* and under conditions when PA layers were exposed to the atmosphere contained gaseous ozone: DPPA *dotted line* and DOPA 18:1 *short dash line*

Preliminary experiments were performed to show differences in action of ozone on saturated and unsaturated lipids. The simplest phospholipids, i.e., phosphatidic acids: saturated DPPA and unsaturated DOPA were taken for this comparison. The results are presented in Fig. 1 where surface pressure isotherms were obtained for not treated (solid lines) and after 6 min exposition of lipid layer to atmosphere containing ozone (short dash and dotted lines). The ozone level reached after 6 min of ozonation was estimated from yield of ozone generator and volume of the chamber 78,900 cm^3^ giving ozone concentration in the range 1–3 ppm. As expected (Castro et al. 2013), ozone does not react with saturated derivative of phosphatidic acid, consequently not causing any modification of its monolayer. Reaction of ozone with unsaturated DOPA (18:1) led to a significant reduction of lipid amount capable of forming an insoluble layer responsible for an increase of interfacial surface pressure (Fig. 1).

Application of ozone from a gas phase produces very severe effects. As it is rather difficult to control ozone level in the atmosphere of the housing of Langmuir trough, all further presented experiments were performed by introducing ozone into aqueous subphase, regulating its concentration by dilution. For ozone concentration determination, samples of subphase containing ozone were taken from a trough at the moment of lipid application.

**Fig. 2:**
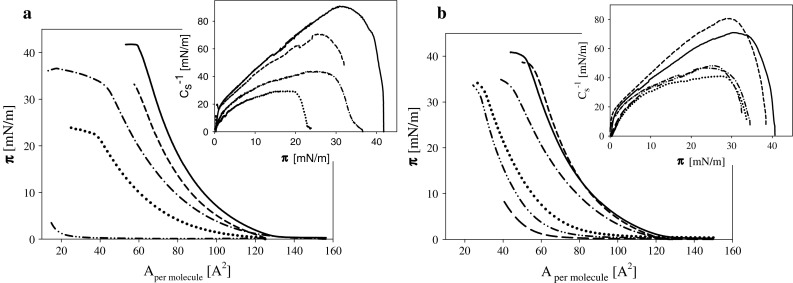
**a**. Surface pressure isotherms of MGDG layers on subphase (1 mM KCl) containing ozone at concentrations [ppm]: 0 *solid*, 0.065 *short dash*, 0.11 *dash*–*dot*, 0.165 *dotted*, 0.34 *dash*–*dot*–*dot lines*. **b**. The same dependencies for DGDG layers on electrolyte solutions containing ozone at concentrations [ppm]: 0 *solid*, 0.0675 *short dash*, 0.11 *dash*–*dot*, 0.19 *dotted*, 0.26 *dash*–*dot*–*dot* and 0.58 *long dash lines*. *Insets* present the dependencies of $C_{\text{s}}^{ - 1}$ on surface pressure *π* for the same systems

Surface pressure isotherms indicate that both studied galactolipids, i.e., DGDG and MGDG form monolayers whose mechanical properties change monotonically with compression degree (molecular packing) without any characteristic phase transitions, structure reorganization (Fig. 2), in agreement with Tazi et al. (1999), Kruk et al. (1992) and Bottier et al. (2007). Maximal packing of galactolipids layers expressed by the area per molecule *A*_min_ corresponding to the pressure at the intercept between the tangents to the collapse plateau and to the end of the pressure–area isotherm were equal to 51 and 60 Å^2^/molecule for DGDG and MGDG, respectively. The higher values of *A*_min_ found for MGDG in comparison to DGDG are in accordance with observations of Bottier et al. (2007). These authors reported for both lipids larger numbers for *A*_min_ (64 and 82 for DGDG and MGDG, respectively). But composition of subphase (0.1 M NaCl) and another distribution of unsaturated fatty acid residues may explain the quantitative disagreement with data obtained in the studies. Compressibility modules of galactolipids layers are rather low (maximal values of *C*_s_^−1^ equal to 91 and 71 mN/m for MGDG and DGDG, respectively) due to their molecular structure (multiple bonds in fatty acid residues and large galactose groups forming polar part of the molecules).

Contact of DGDG and MGDG layers with ozone results in a significant decrease in surface pressure to the degree dependent on ozone concentration (Fig. 2). One can notice that the slopes of surface pressure isotherms are decreasing with ozone concentration indicating an increase of layer compressibility. These changes expressed by the values of *C*_s_^−1^ are presented in insets in Fig. 2.

**Fig. 3:**
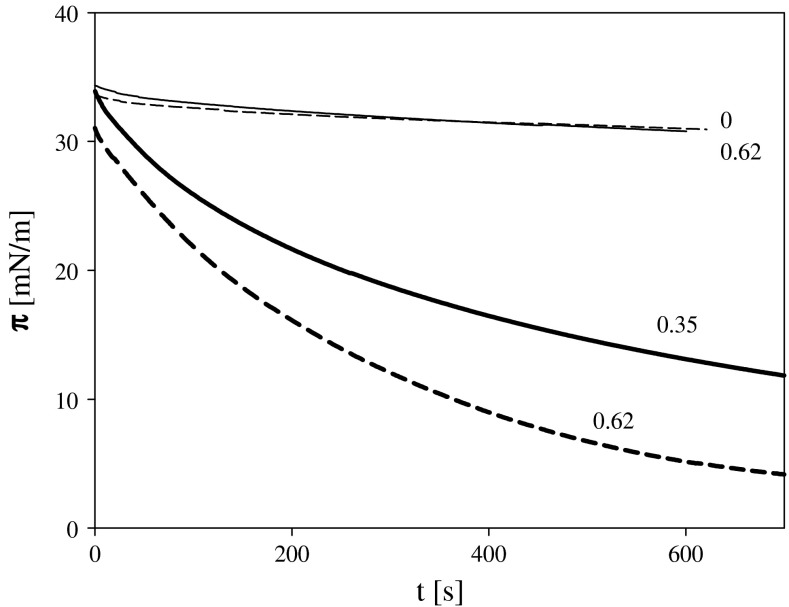
Time dependence of surface pressure of DGDG layers: on 1 mM KCl compressed to *π* equal to 35 mN/m *thin solid line*; layer after contact with solution containing 0.62 ppm of ozone and compressed after reaction to comparable pressure *thin short dash line*; the layer formed by deposition of the amount of DGDG which, at absence of ozone, exhibited the surface pressure **π** equal to 35 mN/m deposited onto electrolyte containing 0.35 *thick solid* and 0.62 ppm of ozone *thick short dash lines*

The impact of ozone can be observed by looking on time dependence of surface pressure under conditions when larger amount of lipids (corresponding to surface pressure of about 30 mN/m, when there was no ozone dissolved in subphase) was applied on solution containing ozone at two concentration levels (Fig. 3). During this experiment, lipid layers were not compressed (constant position of barriers); thus, the observed decrease of surface pressure should be associated with the reaction with ozone. Layers obtained by spreading comparable amount of lipids on the subphase not containing any ozone did not exhibit such significant decrease of surface pressure. Small decrease of π registered under such conditions (probably due to slight lipid dissolution or/and layer reorganization) was comparable with the change observed for the layers of oxidized lipids compressed to the same value of surface pressure. The results presented in Fig. 3 indicate that reaction of galactolipids with ozone leads to a significant drop in the surface pressure, which can be interpreted as due to a decrease in the amount of non-oxidized lipid molecules, building a layer.

**Fig. 4:**
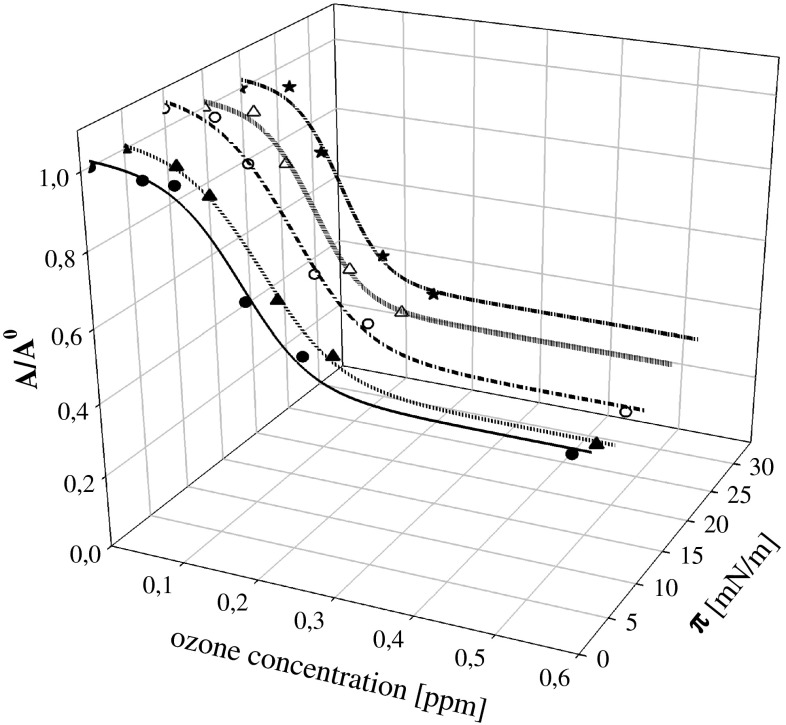
Ozone concentration dependencies of the ratio of *A/A* ^0^ of DGDG layers for various surface pressures: lift-off values *solid line*, subsequent lines correspond to surface pressure values equal to 5, 10, 15, and 20 mN/m

To quantify this effect, we assumed that lipid quantity remaining after the reaction with ozone can be obtained from the molecular area lift-off values *A*_off_ (i.e., molecular areas when the curves *π* = *f*(*A*_m_) take off from the base line). This quantity is often used to characterize the packing density, at which the molecules in the monolayer begin to interact causing a measurable increase in surface pressure (lift-off point is usually identified with area per molecule at which surface pressure increases to the value of 1 mN/m) (Abousalham, et al. 2000; Steinkopf et al. 2008; Thakur et al. 2011; Steinkopf et al. 2012). The ratio of *A*_off_ values found for the layers on ozone-containing subphase to that found for the layer deposited onto ozone-free electrolyte *A*_off_^0^ was taken as a measure of a fraction of lipid left on a surface after reaction. We are aware that this way of estimation is to some extent arbitrary, but provides some appreciation of the effects caused by lipid/ozone reaction. Similar analysis performed for various surface pressure values exhibited similar dependencies on ozone concentration as can be seen in Fig. 4 what supports the assumption made.

To determine some physicochemical properties of the products of lipid-ozone reactions, additional experiments were performed with thin lipid films dried on the wall of the glass tube exposed for 10 min to stream of gas produced by ozone generator. Samples of about 1 mg of lipids were used for formation of dried and next ozonized films. 1–2 ml of 1 mM KCl solution was poured to the tube with oxidized lipid films. After 24 h contact, surface tension and MDA content in the electrolytic extract were measured. For both studied lipids, i.e., MGDG, DGDG solutions contacting their oxidized films exhibited significantly decreased surface tensions (of about 50 mN/m in comparison to 72 mN/m for pure electrolyte). Under the same conditions, electrolyte solutions contacting dried but not ozonized lipid films showed only a slight reduction (by about 1 mN/m) of surface tension. This indicates that products of reaction of studied lipids with ozone are soluble and surface active. Such substantial reduction of surface tension could be registered only because the volume of electrolyte used for extraction of the reaction products from oxidized lipid films was very small (1–2 ml). When taking into account that the volume of subphase in Langmuir trough on which the lipid monolayers were formed was about 200 ml, i.e., almost 200 times larger, the impact of surface activity of oxidation products dissolved in a subphase on surface pressure isotherms was not noticeable.

Analysis of solutions contacting ozonized films of DGDG and MGDG showed the presence of considerable quantities of MDA. Using molar extinction coefficient for MDA/TBA complex equal to 155 mM^−1 ^cm^−1^ (at *λ* = 532 nm), it was found that amount of MDA formed in the reaction of MGDG with ozone was about twice higher than that obtained from DGDG under studied conditions (0.44 and 0.21 mol MDA per mol of lipid, respectively).

Above presented results of ozone-induced changes of properties of galactolipids layers can be interpreted in terms of products of their oxidation. The variety of oxidation products may arise (Brown et al. 2011; Reis and Spickett 2012; Jurkiewicz et al. 2012) starting from full chain length oxidized forms through species of shortened fatty acyl chains to truncated chains. When considering the chemical nature of the groups formed during oxidation these may be saturated and unsaturated aldehydes and hydroxy-aldehydes, carboxylic acids, and hydroxides. Due to a large number of fatty acid residues that have multiple double bonds in studied MGDG and DGDG is to be expected that the reaction products will contain a significant fraction of species of low molecular weight. This explains the disappearance from the surface phase a considerable amount of material capable of forming a monolayer. The finding is in agreement with the results of Mosca et al. (2011) where it has been demonstrated that the size of phosphatidyl choline (PC) liposomes subjected to the oxidation reaction was greatly reduced. MD simulations of oxidized phospholipid (DOPC) monolayers (Khabiri et al. 2012) showed that the role played by low molecular weight oxidation products depends on oxidation conditions. At moderate oxidation, these species can penetrate monolayer causing its swelling. Under stronger oxidation conditions, removal of the short-chain products may lead to disordering the layer.

Oxidation products of shorter chains released to water phase can explain observed decreased surface tension of the solution (of small volume) contacting the oxidized lipid film and significant MDA content in it. It was proved (Mattila 2009) that products of PC oxidation with only partly shortened one acyl chain exhibit also significant surface activity lowering surface tension of their aqueous solutions to about 40 mN/m.

Monolayers which are formed on the surface after oxidation differ from the not oxidized not only in the amount of material, but also in its composition. The oxidized lipid products remained in the layers can strongly modify their properties. Monolayers of both studied lipids: MGDG and DGDG after reaction with ozone exhibit higher compressibilities (as is apparent from the smaller values of *C*_s_^−1^). This is consistent with the literature reports where it was proved experimentally and in MD simulation that species formed in oxidation reaction generally cause a decrease of mixed layer organization leading to

- reversal of oxidized chains (Khabiri et al. 2012)
- increasing head group hydration and mobility (Beranova et al. 2010)
- by changing hydration influencing water penetration in bilayers (Castro et al. 2013)
- changing membrane fluidity (Borst et al. 2000)
- affecting bilayer packing (Megli and Russo 2008).

(All these aspects are summarized in the review of Jurkiewicz et al. 2012.)

**Fig. 5:**
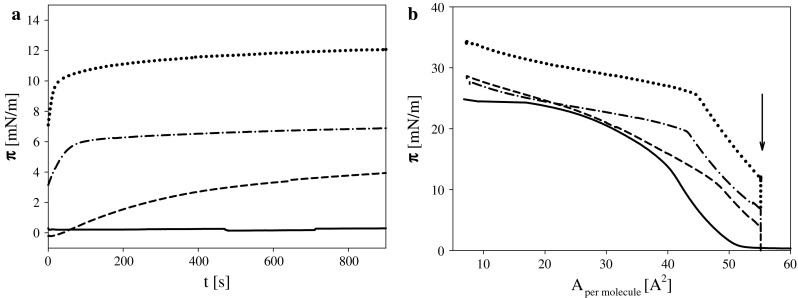
**a** Time dependence of surface pressure of α-tocopherol layers after being deposited on the subphase containing ozone at concentrations [ppm]: 0 *solid*, 0.115 *short dash*, 0.295 *dash*–*dot* and 0.65 *dotted lines*. **b** Surface pressure isotherms for the same systems. The *arrow* shows that step of changes which was presented in part (**a**)

α-Tocopherol—known of its ability to protect biosystems against oxidative stress was chosen to check its influence on the reaction of studied lipids with ozone. In preliminary experiments, the effect of ozone onto the layer of α-tocopherol applied on the electrolyte solution was checked. α-Tocopherol spreads on the aqueous subphase changing its surface pressure (Fig. 5b) in accordance with previous literature reports (Patil and Cornwell 1978; Gzyl-Malcher et al. 2010; Jurak and Miñones Conde 2013). Ozone presence in subphase significantly alters the properties of α-tocopherol layer even without compression. It is clearly visible in time dependence of surface pressure of a layer formed from such an amount of α-tocopherol that, in absence of ozone does not influence surface pressure (Fig. 5a). An increased surface pressure value of α-tocopherol layer spread on acidic subphase (manifested as a shift of surface pressure isotherms in direction of higher values of area per molecule) was registered by Patil and Cornwell (1978) where tocopherol was oxidized by air.

To find the cause of the such substantial increase of surface pressure, dried films of α-tocopherol: not exposed and after 10 min exposition to ozone in gas phase were dispersed in 1 mM KCl using ultrasound dispergator. The electrophoretic mobility of such particles was measured using Malvern Zetasizer. It occurred that particles obtained from ozonized film exhibited much higher mobility which after recalculation onto zeta potential (according to Smoluchowski equation) gave an increase of its absolute value by about 65 mV (zeta potential of α-toc particles in 1 mM KCl was equal to −12.4 ± 1.0 mV; of particles formed from oxidized α-toc film −78.3 ± 4.23 mV). Thus, observed increase in surface pressure may be attributed to the higher charge of layers containing products of α-tocopherol oxidation what obviously increases electrostatic repulsive interactions. It is known from the literature (Patil and Cornwell 1978; Csallany and Ha 1992; Kamal-Eldin and Appelqvist 1996; Liebler et al. 1996; Leray et al. 1998; Enami et al. 2009) that oxidation of α-tocopherol may give different products depending on: type of oxidizing agent, protic or aprotic character of the environment, pH of polar media. Two stable products of α-tocopherol oxidation, i.e., α-tocopherolquinone (the major metabolite in vivo) and dimer, formed by quenching reaction of two tocopherol radicals, seem to be most important. Quinone class compounds undergo various redox equilibriums which may be accompanied by dissociation of some their forms (Guin et al. 2011; Rao and Hayon 1973). The appearance of the ionized species in a layer of oxidized tocopherol will, at least partially, explain an increase in surface pressure registered at ozone presence.

**Fig. 6:**
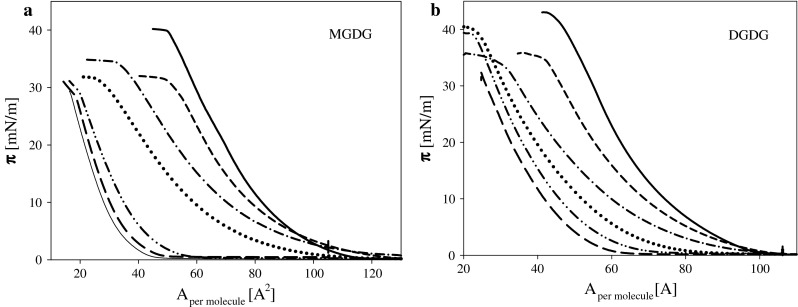
**a** Surface pressure isotherms of mixed MGDG:tocopherol (3:1 w/w, molar ratio 1.7:1) layers on subphase (1 mM KCl) containing ozone at concentrations [ppm]: 0 *solid*, 0.055 *short dash*, 0.13 *dash*–*dot*, 0.19 *dotted*, 0.28 *dash*–*dot*–*dot*, 0.52 *long dash*, 0.89 *thin solid lines*. **b** The same dependencies for mixed DGDG:tocopherol (3.6:1 w/w, molar ratio 1.7:1) layers on electrolyte solutions containing ozone at concentrations [ppm]: 0 *solid*, 0.06 *short dash*, 0.16 *dash*–*dot*, 0.29 *dotted*, 0.46 *dash*–*dot*–*dot* and 0.59 *long dash lines*

**Fig. 7:**
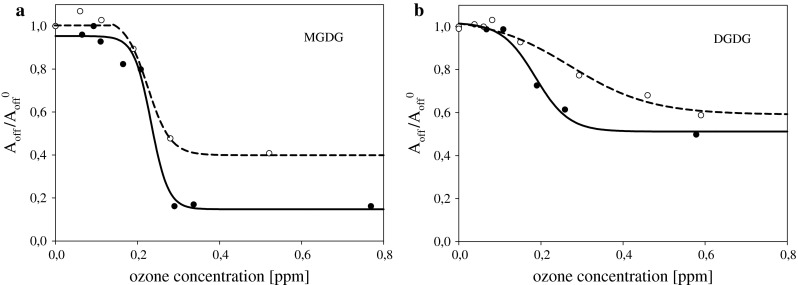
**a** Ozone concentration dependencies of the ratio of *A*_off_/*A*_off_^0^ of MGDG *solid line* and of mixed MGDG:tocopherol (3:1 w/w, molar ratio 1.7:1) layers *dashed line*. **b.** The same dependencies for the systems: DGDG *solid line* and for the mixture DGDG:tocopherol (3.6:1 w/w, molar ratio 1.7:1) *dashed line*

To verify the protective effect of α-tocopherol, the mixtures of compositions: MGDG:tocopherol 3:1 w/w and DGDG:tocopherol 3.6:1 w/w (what corresponds to molar ratio 1.7:1) were used for monolayer formation on subphases containing various ozone concentrations (Fig. 6). The same way of data analysis as used for single galactolipid layers was applied to their mixtures with tocopherol. The results are presented in Fig. 7 in the form of the dependencies of the ratio of molecular area lift-off values for layers on subphases containing defined amount of ozone relative to the value for layers formed on pure electrolyte solution on ozone concentration. One can see that MGDG is much more sensitive to ozone presence which leads to almost 90 % decrease in an amount of molecules capable for layer formation after contact with ozone at concentrations above 0.3 ppm, whereas about 50 % of DGDG and its oxidation products stayed at interface forming a monolayer. This finding is in agreement with a significantly higher level of MDA found in the samples contacting oxidized film of MGDG as compared to DGDG. As was discussed above, significant loss of molecules capable of forming monolayers can be linked to oxidation products which, at least partially can be transferred to the aqueous phase. Sabatini et al. (2006) and Khabiri et al. (2012) on the basis of experimental results and MD simulations postulate that reorientation of the oxidized lipid chains, enhanced infiltration of water into the lipid region and penetration of the short-chain oxidation products between lipid molecules may eventually result in solubilization of the oxidized phospholipids.

Different impact of ozone on layers of MGDG and DGDG, despite similar number of double bonds in their fatty acid residues, can be linked to the protective effect of galactose group. Filek et al. (2010b) and Łabanowska et al. (2012) postulated that under oxidizing conditions sugar units can serve as locations for free electrons forming there carbohydrate radicals of low reactivity which, in turn, can additionally work as a “trap” for other free radicals. This hypothesis explains the greater resistance to oxidation of DGDG having two galactose groups compared to MGDG containing one such group.

The effect of ozone onto mixed layers of galactolipid:α-tocopherol at molar ratio 1.7:1 demonstrated more effective protection of MGDG than DGDG layers by α-tocopherol under studied conditions. This observation can be explained by discussing the role of layer organization onto protective ability of tocopherol. According to (Marquardt et al. 2013) location of all participants of oxidation reaction, i.e., oxidants, antioxidants, and oxidation substrates at hydrophobic–hydrophilic region of the membrane is especially favorable for interface reduction of ROS and lipid radicals. This condition is important not only for the lipid oxidation but also for the effective operation of antioxidants. One can imagine that the two galactose groups in DGDG, which by virtue of their polarity can locate in this part of the membrane, may create steric hindrance to the operation of tocopherol. However, this hypothesis should be confirmed by further experimental studies and MD modeling.

## Main Conclusions

1. DGDG layers respond to ozone presence in aqueous solutions at concentrations higher than 0.1 ppm. Based on the assumption that the surface pressure decrease found for layers exposed to ozone may be associated with oxidation products exiting a layer, the molecular area lift-off values were used for estimation of loss of molecules from the surface. Reaction DGDG with ozone at higher concentrations results in an approximately 50 % decrease of molecules capable to form monolayers.
2. Reaction of MGDG with ozone produces similar effects, however, the presence of higher ozone concentrations in subphase resulted in much stronger (above 80 %) elimination of MGDG molecules responsible for layer formation. Consequently, a higher amount of MDA was found in reaction products of MGDG with ozone than in case of DGDG oxidation.
3. Oxidized films of both studied galactolipids release to aqueous phase soluble reaction products which occurred to be surface active—diminishing effectively surface tension of electrolyte solutions (of small volume) used for extraction.
4. Differences in impact of ozone onto MGDG and DGDG layers can be associated with the protective action of galactose groups which can serve as locations for free electrons forming there low reactive carbohydrate radicals becoming a “trap” for other free radicals (as suggested in (Filek et al. (2010b) and Łabanowska et al. (2012)).
5. Reaction with ozone causes noticeable increase of surface pressure οf α-tocopherol layer. This effect has been linked to a significant increase of absolute values of the zeta potential of particles formed from oxidized tocopherol film. The observation was explained in terms of quinone-type products of α-tocopherol oxidation, potentially undergoing various redox equilibriums accompanied by dissociation of some their forms (Guin et al. 2011; Rao and Hayon 1973). Ionized species condition the occurrence of electrostatic repulsions which in turn explain the experimentally detected surface pressure rise.
6. In case of MGDG, the presence of α-tocopherol at studied lipid: α-toc ratio does not shift the ozone sensitivity threshold (being still at about 0.2 ppm) but strongly influences the amount of lipid capable of forming a layer left under higher ozone concentrations increasing it from 16 to about 40 %. The effect of α-tocopherol on DGDG layer oxidation is weaker increasing by approximately 10 % the fraction of molecules forming the layer after reaction with ozone at higher concentrations. The different protective impact of α-tocopherol onto DGDG and MGDG was discussed basing on the work of Marquardt et al. (2013) where large role for studied processes was assigned to the location of all participants of oxidation reaction at hydrophobic–hydrophilic part of an interfacial region. Under such assumption second galactose group of DGDG may create steric obstacle for the tocopherol protective operation.
7. The presented results allow to explain the observed changes in the composition of lipids (reduction of galactolipids) in the chloroplast membranes under oxidative stress conditions. The consequence of these changes is an alteration of physicochemical parameters characterizing the mechanical and electrical properties responsible for fluidity and transport processes to and from cells and organelles. A modification of the membrane structure caused by the loss of galactolipids and formation of oxidation products will possibly affect also the protein–lipid interaction and thereby the location and activity of the bound proteins.
